# Revolutionizing Breast Healthcare: Harnessing the Role of Artificial Intelligence

**DOI:** 10.7759/cureus.50203

**Published:** 2023-12-08

**Authors:** Arun Singh, Shivani B Paruthy, Vivek Belsariya, Nemi Chandra J, Sunil Kumar Singh, Sri Saran Manivasagam, Sushila Choudhary, M Anil Kumar, Dhananjay Khera, Vaibhav Kuraria

**Affiliations:** 1 General Surgery, Vardhman Mahavir Medical College and Safdarjung Hospital, New Delhi, IND; 2 Surgical Oncology, Vardhman Mahavir Medical College and Safdarjung Hospital, New Delhi, IND; 3 General Surgery, Maulana Azad Medical College, New Delhi, IND

**Keywords:** breast cancer management, artificial intelligence, breast cancer, breast screening, deep learning artificial intelligence

## Abstract

Breast cancer has the highest incidence and second-highest mortality rate among all cancers. The management of breast cancer is being revolutionized by artificial intelligence (AI), which is improving early detection, pathological diagnosis, risk assessment, individualized treatment recommendations, and treatment response prediction. Nuclear medicine has used artificial intelligence (AI) for over 50 years, but more recent advances in machine learning (ML) and deep learning (DL) have given AI in nuclear medicine additional capabilities. AI accurately analyzes breast imaging scans for early detection, minimizing false negatives while offering radiologists reliable, swift image processing assistance. It smoothly fits into radiology workflows, which may result in early treatments and reduced expenditures. In pathological diagnosis, artificial intelligence improves the quality of diagnostic data by ensuring accurate diagnoses, lowering inter-observer variability, speeding up the review process, and identifying errors or poor slides. By taking into consideration nutritional, genetic, and environmental factors, providing individualized risk assessments, and recommending more regular tests for higher-risk patients, AI aids with the risk assessment of breast cancer.

The integration of clinical and genetic data into individualized treatment recommendations by AI facilitates collaborative decision-making and resource allocation optimization while also enabling patient progress monitoring, drug interaction consideration, and alignment with clinical guidelines. AI is used to analyze patient data, imaging, genomic data, and pathology reports in order to forecast how a treatment would respond. These models anticipate treatment outcomes, make sure that clinical recommendations are followed, and learn from historical data. The implementation of AI in medicine is hampered by issues with data quality, integration with healthcare IT systems, data protection, bias reduction, and ethical considerations, necessitating transparency and constant surveillance. Protecting patient privacy, resolving biases, maintaining transparency, identifying fault for mistakes, and ensuring fair access are just a few examples of ethical considerations. To preserve patient trust and address the effect on the healthcare workforce, ethical frameworks must be developed. The amazing potential of AI in the treatment of breast cancer calls for careful examination of its ethical and practical implications. We aim to review the comprehensive role of artificial intelligence in breast cancer management.

## Introduction and background

Breast cancer has the highest incidence and second-highest mortality rate among all cancers [1]. The significance of breast cancer management lies in its potential to safeguard lives, boost the quality of life, and minimize the impact of this widespread and potentially deadly disease. Early discovery is the first step in the effective management of breast cancer, and it can increase survival chances. The likelihood of successful treatment is increased by routine screening tests and early diagnosis [2]. A reduced mortality rate is a result of timely management and appropriate medical care. Breast cancer management endeavors to improve the patient's quality of life in addition to curing the disease. This means reducing adverse effects to a minimum while maintaining breast function and body image [3]. Every case of breast cancer is distinct, and management entails modifying therapy to fit each patient depending on their unique diagnosis, stage, and previous medical history. Treatment results are improved by this individualized approach. The emotional and psychological well-being of patients is taken into consideration during breast cancer care. It offers assistance in managing the stress, anxiety, and emotional difficulties brought on by the condition [4]. Current medical research and advancements are driven by breast cancer management. This research promotes the creation of novel therapies, technologies, and methodologies for the more effective management of breast cancer. By lowering the overall toll of the disease on healthcare systems and society, effective breast cancer management enhances public health. Furthermore, it supports initiatives for prevention, early detection, and awareness about breast cancer [5].

Nuclear medicine has used artificial intelligence (AI) for over 50 years, but more recent advances in machine learning (ML) and deep learning (DL) have given AI in nuclear medicine additional capabilities. Tasks such as identification and quantification from radiomic images are improved by ML, particularly when convolutional neural networks (CNNs) are used. This evolution has impacted medical research and practice, necessitating the knowledge of AI principles by specialists and allowing for improved problem-solving and a variety of applications, from quality control to data analysis [6]. Convolutional neural networks and deep learning are key components of computer-aided radiological image processing, which is essential for clinical workflows from diagnosis to treatment. Their use across a range of anatomical regions and tasks has grown significantly in recent years. Deep learning refers to a composite of numerous simple functions that are deeply nested, mostly linear combinations such as convolutions, scalar non-linearities, and moment normalizations, parameterized by variables. Medical image analysis via deep learning pipelines has some basic elements, but due to particular clinical requirements and data formats, they also need domain-specific modifications [7]. Deep learning pipelines for medical image analysis are made up of numerous interconnected parts: (A) data augmentation, (B) a network architecture, which is defined as an amalgam of many simple functions, (C) a fast computational framework for optimized performance and inference, (D) metrics for assessing performance during training and inference, (E) categorization of data into training, testing, and validation sets, (F) randomized sampling during training, and (G) image data loading and sampling [7]. The breakthroughs in imaging, digital pathology, diagnosis, and treatment of breast cancer made possible by the use of AI approaches are the focus of this article, along with some of the accompanying downsides.

## Review

Artificial intelligence for early detection through imaging

Mammograms and other breast imaging scans can be accurately analyzed by AI systems. They can pick up on subtle abnormalities that can be difficult for human radiologists to pick up on, such as microcalcifications or early-stage tumors. False-negative findings, which happen when cancer is present, but not diagnosed, can be decreased with AI. AI can improve the likelihood of detecting tumors in their early stages by consistently scanning radiographs for potential cancer signs [3]. AI is used by computer-aided detection (CAD) systems to automatically point to pathological spots on medical images such as mammograms. Radiologists can then meticulously inspect the areas that have been marked, decreasing the possibility of overlooking abnormalities. Rapid evaluation of huge quantities of medical images using AI enables quicker interpretation and diagnosis. This is especially helpful in busy medical facilities where there could be a shortage of time for each case [8]. AI systems operate consistently and are unaffected by variables such as human fatigue, distraction, or changes in human interpretation. This constancy ensures that no potential pathologies are disregarded. The workflow in radiology can easily incorporate artificial intelligence (AI) technologies. They benefit radiologists by offering greater information during the evaluation of radiological images, enabling them to make more precise assessments [9]. By learning from an extensive collection of radiological image data, AI models can continuously enhance their performance. As they examine more cases, they excel at detecting early indications of breast cancer. AI can help with classifying patients based on risk profiles, ensuring that individuals who are at greater risk receive more frequent screenings and vigilant monitoring. AI-assisted early diagnosis can result in earlier interventions and less aggressive therapies, which may lower the overall cost of breast cancer treatment [10].

Artificial intelligence in pathological diagnosis

Digital pathology photographs of breast tissue slides can be accurately evaluated by AI systems. They enable pathologists to diagnose cancer through the recognition of cellular structures, anomalies, and malignant areas [11]. The utilization of AI can accelerate pathologists' tasks and minimize the likelihood of human error by automatically recognizing and delineating tumor boundaries on pathology slides. Using AI, it is possible to precisely count the number of mitotic figures present in tissue samples, a crucial component in assessing breast cancer's aggressiveness. In order to help pathologists evaluate the severity of the disease, AI algorithms can categorize breast cancer subtypes (e.g., estrogen receptor (ER)-positive and human epidermal growth factor receptor 2 (HER2)-positive) and provide tumor grades. AI produces consistent outcomes, minimizing pathologists' inter-observer variability and assuring standardized diagnoses [12]. Through the use of AI, the review process can be expedited, allowing pathologists to concentrate on difficult slides and complex cases. By spotting errors, artifacts, or slides with poor image quality, AI algorithms can potentially make sure that only reliable data is used for diagnosis. AI can forecast biological biomarkers (such as the Ki-67 proliferation index) from pathology slides to help with treatment decisions and prognosis [13]. In order to provide a complete picture of the patient's history and help with planning treatment, AI systems can integrate pathological findings with patient records. AI makes it easier to analyze pathology data on a large scale, which helps researchers explore new biomarkers, responses to treatments, and disease mechanisms. By enabling pathologists to review and confer on cases remotely, AI-powered telepathology systems expand access to knowledge and break down geographical barriers. Pathology students can benefit from using AI-assisted tools as excellent resources for learning to help them precisely recognize and diagnose breast cancer [14].

Artificial intelligence in risk assessment

By examining numerous patient-related components and medical data, artificial intelligence (AI) facilitates the risk assessment of breast cancer. A person's distinctive combination of risk factors, such as age, family history, genetics, lifestyle, and medical history, is taken into account by AI models. AI can offer personalized risk evaluations for breast cancer by reviewing these components collectively. In order to identify those with a higher hereditary propensity to breast cancer, AI can analyze genetic data, such as breast cancer gene 1 (BRCA1) and BRCA2 mutations. This knowledge facilitates the development of screening and preventative programs [15]. For people at higher risk, AI-driven risk assessment models may suggest more frequent evaluation or additional imaging, enabling the early diagnosis of breast cancer. In order to determine total risk, AI can take into account environmental variables such as radiation exposure in addition to lifestyle facets such as alcohol intake, smoking, and physical exercise. In order to give a comprehensive risk assessment, AI incorporates a variety of medical data sources, including electronic health records (EHRs), radiological scans, pathology reports, and genetic screening results. In order to maintain the precision of risk predictions over time, AI models can continuously update risk evaluations as and when new data becomes available [15]. AI offers risk assessment tools and suggestions to healthcare providers, encouraging them to make decisions regarding screening, prevention, and risk reduction measures. AI can produce comprehensive risk assessment reports for patients, enhancing patient education and joint decision-making with healthcare professionals. AI-driven models for risk evaluation can help identify novel risk markers and enhance existing research activities on breast cancer risk factors. AI can evaluate data at the population level to find trends and risk factors within particular populations, assisting public health authorities in creating tailored prevention strategies, such as the National Health Service (NHS) Breast Screening Program [16].

AI for personalized treatment recommendations

AI combines genetic data, such as mutations and biomarkers, with clinical details, such as the individual's age, cancer stage, tumor characteristics, and comorbidities. The most efficient treatment alternatives are found through this comprehensive analysis of the data. To forecast how a patient will likely respond to various treatment methods, AI algorithms evaluate historical patient data. This aids oncologists in making the least toxic and most successful treatment decisions. AI assesses genomic data from tumor samples to find particular genetic mutations or variations that may react to targeted therapy. This makes it possible to offer precise medication based on the genetic makeup of the patient. AI takes into account potential drug interactions and adverse effects, as well as the compatibility of various treatments and the patient's medication history. This lowers adverse consequences and increases therapeutic effectiveness [17]. The most recent therapeutic recommendations and scientific discoveries are programmed into AI systems. They offer treatment suggestions that are in line with evidence-based practices, ensuring that patients get the best possible treatment. AI aids in the creation of a customized treatment strategy that may comprise surgery, chemotherapy, radiation therapy, immunotherapy, or an amalgamation of these. It also takes into account the best possible sequence and timing of therapies. Throughout treatment, AI keeps track of the patient's development and modifies its recommendations in conjunction with emerging data. This enables prompt treatment plan revisions in response to evolving situations. AI-generated treatment recommendations enable patients and healthcare professionals to adopt shared, well-informed choices. Patients are better equipped to comprehend the treatment options and possible implications. In order to provide access to cutting-edge therapy for qualified patients, AI can find appropriate clinical trials that offer experimental treatments in line with the patient's profile. AI takes cost-effectiveness into account while recommending treatment modalities, assisting patients and healthcare systems in making appropriate resource allocation decisions [18].

AI in treatment response prediction

Medical records, treatment strategies, and patient outcomes are all analyzed by AI models using historical patient data. By observing how patients with comparable conditions in the past have responded to various therapies, AI develops trends and associations that can be used to forecast how new patients will respond to various therapies. AI can review diagnostic images such as MRI, CT, or PET scans to evaluate how a tumor's size, shape, and density evolve during the course of treatment. This helps to evaluate the extent to which the tumor is responding to treatment. Data from genomic profiling and biomarker analyses is analyzed by AI. It pinpoints genetic changes or mutations that could affect a patient's reaction to treatment and offers information about targeted therapeutics [19]. AI evaluates changes in tumor features, including tumor grade, cell proliferation, and molecular markers, which may predict treatment response, from pathology reports and tissue samples. AI regularly evaluates a patient's treatment progress. As updated data becomes available, forecasts are adjusted by comparing the patient's actual response to the projected response. AI matches known clinical guidelines and evidence-based practices to treatment response predictions. When results differ from expectations, messages are sent to healthcare professionals, urging them to reevaluate their treatment options. AI uses algorithms based on machine learning, such as decision trees, random forests, support vector machines, or neural networks, to evaluate complex correlations between patient data and treatment outcomes [20]. AI's evaluation of the effectiveness of a treatment can take into account patient-reported outcomes and responses concerning adverse effects, symptoms, and quality of life. To provide a comprehensive picture of a patient's reaction to treatment, AI integrates data from several sources, such as electronic health records, imaging systems, genetic testing platforms, and clinical trial databases [18].

Artificial intelligence-mediated de-escalating strategies

De-escalation tactics are specifically designed for distinct subgroups with breast cancer. Tumor type, stage, biomarker status (e.g., hormone receptor or HER2 status), and patient preferences are frequently taken into account in selection criteria. AI-mediated de-escalation may entail the use of specific drugs to selectively target cancer cells, such as hormone therapy or HER2-targeted medications, reducing the need for more strenuous therapies such as chemotherapy [21]. Instead of a full mastectomy, breast-conserving surgery (lumpectomy) followed by radiation therapy may be recommended in many situations. For some people, this method retains breast tissue and produces comparable results. In order to lessen the burden of radiation therapy on the patient's everyday life, accelerated partial breast irradiation (APBI) or hypofractionated radiation therapy may be employed [22].

To reduce the risk of lymphedema and shoulder function difficulties, sentinel lymph node biopsy (SLNB) may be used instead of axillary lymph node dissection (ALND) for certain people with early-stage breast cancer. De-escalation tactics emphasize avoiding excessive treatment. This means avoiding subjecting patients to medical interventions that might provide only marginal benefit but have serious adverse effects. AI-mediated active surveillance, in which patients are continually tracked with routine imaging and clinical examinations while definitive treatment is postponed, may be an option for some low-risk, early-stage breast tumors [23]. De-escalation seeks to minimize the intensity of the treatment given to the patient, but it is vital to maintain equivalent rates of survival and ensure that the patient's long-term prognosis is not jeopardized. Long-term follow-up is necessary for patients receiving de-escalation techniques in order to track treatment effectiveness and identify any disease progression or recurrence [24].

Artificial intelligence-mediated patient stratification

AI aids in the creation of a customized treatment strategy through a comprehensive analysis of data. Estrogen receptor (ER) and progesterone receptor (PR) status are two hormone receptors that are frequently used to classify breast cancer. Hormonal therapy may help patients with tumors that express hormone receptors. The status of the human epidermal growth factor receptor 2 (HER2/neu) is another important consideration. Targeted treatments such as trastuzumab (Herceptin) may be effective for treating HER2-positive breast tumors [25]. Patients who could have a higher hereditary risk of developing breast cancer or who might benefit from particular treatments or preventive measures might be identified by genetic testing, such as finding BRCA1 and BRCA2 mutations. A patient's lifetime risk of acquiring breast cancer can be calculated using a variety of risk assessment models, including the Tyrer-Cuzick model and the Gail model. This knowledge can direct screening and preventative methods [26].

Ethical concerns and limitations

AI depends on patient data, including potentially private medical records and photos. It is crucial to protect patient privacy and make sure that robust information security measures are in place to safeguard it against breaches and unauthorized access. The deployment of AI in healthcare should be explicitly disclosed to patients, who should also give their informed consent for data sharing and analysis. Transparency regarding the impact of AI on diagnosis, treatment, and data utilization is crucial [27]. Clinicians may find it difficult to comprehend how AI systems generate suggestions due to their complexity. Building confidence among medical professionals necessitates making sure AI systems are clear and understandable. It might be difficult to assign accountability for mistakes or inaccurate recommendations given by AI systems. Establishing clear protocols for responsibility and culpability is necessary. AI implementation needs to be done in a way that does not make disparities in medical care worse. The fair access of underprivileged and marginalized communities to AI-enhanced healthcare should be emphasized [28].

It is crucial to strike a balance between clinical autonomy and AI's function. The final word in treatment selection should rest with clinicians, with AI acting as a useful guidance and assistance tool. The expense of applying AI in breast cancer management may have an impact on how healthcare organizations allocate resources. Healthcare priorities in general should be taken into account when making investment decisions in AI [29]. To keep them accurate and up-to-date with the most recent medical information, AI systems should be regularly updated and evaluated. Invalid recommendations could result from ignoring this responsibility. It is essential to create and abide by ethical frameworks and standards for AI use in the treatment of breast cancer. These frameworks ought to take patient autonomy, data ethics, and fairness into consideration. It is crucial to make sure that patients believe in AI-driven guidance and feel empowered to take charge of their treatment. To create patient trust, open communication and joint decision-making are crucial. Healthcare personnel may get worried about loss of employment or role changes as a result of the adoption of AI. The impact on the healthcare workforce should be taken into account while making ethical decisions [30].

There are limitations that accompany AI's advancements in the treatment of breast cancer. Incomplete or biased training data can produce biased algorithms. Clinical decision-making is hampered by the lack of transparency in complex AI models such as deep learning neural networks. It is imperative to preserve sensitive patient data, which necessitates significant investments in data protection. Lack of clinical verification may jeopardize credibility. Over-dependence on AI may result in mistakes that require human intervention. Concerns about the influence on the workforce, ethics, legislation, and standardization are common, as are implementation costs and problems with data standards. We must acknowledge and tackle these constraints to integrate AI responsibly in the treatment of breast cancer [31,32].

## Conclusions

Artificial intelligence has revolutionized breast cancer management by enhancing early detection, diagnostic precision, risk assessment, personalized treatments, and predictive analytics. These advancements have improved patient care, reduced treatment side effects, and fostered patient-centered decision-making. Ethical considerations and effective clinical adoption are vital to harness the full potential of AI in breast cancer management, ensuring that it aligns with the broader goals of improving public health and healthcare outcomes.
